# Human IgG response to a salivary peptide, gSG6-P1, as a new immuno-epidemiological tool for evaluating low-level exposure to *Anopheles *bites

**DOI:** 10.1186/1475-2875-8-198

**Published:** 2009-08-13

**Authors:** Anne Poinsignon, Sylvie Cornelie, Fatou Ba, Denis Boulanger, Cheikh Sow, Marie Rossignol, Cheikh Sokhna, Badara Cisse, François Simondon, Franck Remoue

**Affiliations:** 1 UR016-IRD (Institut de Recherche pour le Développement), Caractérisation et Contrôle des Populations de Vecteurs, 911 avenue Agropolis, BP 64501, 34394 Montpellier Cedex 5, France; 2 UR077-IRD, Campus International IRD-UCAD, route des Pères Maristes, BP 1836, Dakar, Sénégal; 3 UR024-IRD, Montpellier, France and Campus International IRD-UCAD, Dakar, Sénégal; 4 Département de parasitologie, Université Cheikh Anta Diop, Dakar, Sénégal

## Abstract

**Background:**

Human populations exposed to low malaria transmission present particular severe risks of malaria morbidity and mortality. In addition, in a context of low-level exposure to *Anopheles *vector, conventional entomological methods used for sampling *Anopheles *populations are insufficiently sensitive and probably under-estimate the real risk of malaria transmission. The evaluation of antibody (Ab) responses to arthropod salivary proteins constitutes a novel tool for estimating exposure level to insect bites. In the case of malaria, a recent study has shown that human IgG responses to the gSG6-P1 peptide represented a specific biomarker of exposure to *Anopheles gambiae *bites. The objective of this study was to investigate if this biomarker can be used to estimate low-level exposure of individuals to *Anopheles *vector.

**Methods:**

The IgG Ab level to gSG6-P1 was evaluated at the peak and at the end of the *An. gambiae *exposure season in children living in Senegalese villages, where the *Anophele*s density was estimated to be very low by classical entomological trapping but where malaria transmission occurred during the studied season.

**Results:**

Specific IgG responses to gSG6-P1 were observed in children exposed to very low-level of *Anopheles *bites. In addition, a significant increase in the specific IgG Ab level was observed during the *Anopheles *exposure season whereas classical entomological data have reported very few or no *Anopheles *during the studied period. Furthermore, this biomarker may also be applicable to evaluate the heterogeneity of individual exposure.

**Conclusion:**

The results strengthen the hypothesis that the evaluation of IgG responses to gSG6-P1 during the season of exposure could reflect the real human contact with anthropophilic *Anopheles *and suggest that this biomarker of low exposure could be used at the individual level. This promising immuno-epidemiological marker could represent a useful tool to assess the risk to very low exposure to malaria vectors as observed in seasonal, urban, altitude or travellers contexts. In addition, this biomarker could be used for the surveillance survey after applying anti-vector strategy.

## Background

The re-emergence of mosquito-borne diseases represents a major public health problem in developing and developed countries, highlighting the need to develop new tools to assess the risk of disease transmission. Malaria has a broad range of different epidemiological profiles, depending on the distribution and vectorial capacity of the mosquito vectors, environmental conditions, and the degree of protective immunity acquired by the exposed population. Control strategies need to be formulated according to transmission and exposure patterns, and the behaviour of the relevant *Anopheles *species.

The WHO reported that 29% of the world's population lives in areas where the level of malaria transmission is low [1]. This mainly corresponds to strictly seasonal transmission, or highland (> 1,500 m), arid (< 1,000 mm rainfall/year) and urban areas. For example, urban malaria is now becoming a serious public health problem in several rapidly growing African cities (largely due to migration from the countryside)[2]. Despite exposure to low numbers of *Anopheles *vectors, people in these settings could be at a high risk of malarial morbidity and mortality because protective immunity is acquired so slowly. Currently, strategies for malaria surveillance and control are of limited efficiency in these epidemiological low-level contexts. Assessing the risk of malaria is based on entomological parameters (the entomological inoculation rate [EIR]) and parasitological methods (*Plasmodium *density in human blood). In a context of low transmission, parasitological methods are limited and need large-scale field evaluation. The evaluation of *Anopheles *density using entomological methods (traps, household/indoor sprayings, human-landing catches, etc.), the first step in an EIR assessment, is not sensitive enough to permit estimation of low-level exposure to *Anopheles*: the total number of *Anopheles *collected is too low to estimate real exposure and thus the risk of malaria transmission. In addition, such methods are mainly applicable at the population level and do not enable the evaluation of the heterogeneity of individual exposure. A simple, rapid, highly sensitive tool is, therefore, needed to evaluate low-level exposure to *Anopheles *bites and the risk of malaria in such populations.

It has been proposed that the level of exposure to vector bites could be evaluated by measuring the antibody (Ab) response to arthropod saliva in exposed populations [3,4]. The salivary proteins of haematophagous insects have a dual role to facilitate blood-feeding: their pharmacological activities counteract human defense mechanisms (inflammation and blood clotting); and their immunological activities modulate the human host's immune response [5,6]. Some of these salivary proteins are immunogenic and induce a specific Ab response [7,8]. Several studies have shown that the Ab response specific to salivary proteins could be used as a marker of exposure to vector-borne diseases in individuals bitten by arthropod vectors, e.g. ticks [9], sandflies [10], *Triatoma *[11], *Glossina *[12] and *Aedes *[13,14]. As far as *Anopheles *spp. and malaria transmission are concerned, early epidemiological studies showed that individuals living in malaria-endemic areas developed Ab responses specific to salivary proteins that represent a marker of exposure to *Anopheles *bites [15,16].

Recently, a study has identified the SG6 salivary protein as an encouraging candidate as serological marker of exposure. The SG6 was previously reported i) specific to the *Anopheles *genus [17,18] and ii) antigenic [19]. By a step-by-step approach, coupling bioinformatic and immuno-epidemiological approaches, the gSG6-P1 peptide (derived from the gSG6 salivary protein of *Anopheles gambiae*) was defined and validated as a potential immuno-epidemiological marker specific to *An. gambiae *exposure [20]. This peptide appears to meet several of the requirements expected of such an exposure marker. First, it appears to be specific to the *Anopheles *genus and would not be expected to cross-react with epitopes from other proteins (from the main *Diptera *species or pathogens). Second, it is synthetic and can, therefore, be used to develop a reproducible immunological assay. Third, it elicits a specific Ab response, which correlates positively with the level of exposure to *An. gambiae *bites.

The aim of this work was to evaluate the pertinence of the gSG6-P1 biomarker in areas where this is a low level of exposure to *An. gambiae*. To this end, the study was conducted in Senegal where malaria transmission is strictly seasonal and especially in villages where few or no *An. gambiae *were collected by classical entomological methods but where a malaria transmission occurred. This study focused on IgG Ab responses to gSG6-P1 peptide in children having had minimal exposure to *An. gambiae*, between the peak and the end of the transmission season.

## Methods

### Study population

The study was conducted in Niakhar, a rural district of central Senegal. This area is characterized by a dry savannah with a rainy season from July to October. This area is typical of the Sahel and sub-Sahel regions of Africa, where malaria is unstable with most *Plasmodium falciparum *transmission occurring between September and November [21].

Sera were available from a clinical trial on seasonal intermittent preventive treatment (IPT) for prevention of malaria performed in 2002 in children aged six weeks to sixty months [22]. Pairs of sera from a sub-sample of these children were available from both the peak (September) and the end (December) of the exposure season to *Anopheles*, as previously described [15]. In September and in December, thick blood smears were collected from each individual involved in the clinical trial. *P. falciparum *prevalences (positive thick blood smears) were assessed for all children living in the six studied villages (n_September _= 403 and n_December _= 388), assuming an average white blood-cell count of 8,000 per μL. In addition, the outcome of malaria morbidity from September to December was reported from all children residing in the six studied villages; clinical malaria was defined as an axillary temperature ≥ 38°C and the presence of *P. falciparum *with a density > 3,000 parasites/μL of blood (thick smear) [15,22]. Sixty-one children from the placebo group were selected as a sub-sample for the immunological analysis in both September and December 2002.

Both the trial on malaria treatment and this study followed ethical principles as stipulated in the Edinburgh revision of the Helsinki Declaration, and were approved by the Ethics Committees of the Ministry of Health of Senegal (August 2002 and May 2003, respectively) and of the IRD (Institute of Research for the Development) (January 2004). The malaria treatment trial was approved by the Ethics Committee of the London School of Hygiene and Tropical Medicine in June 2002.

### Entomological data

Entomological data were collected every month between September and December 2002 in the six studied villages in the Niakhar area. Indoor samplings by light traps (CDC miniature light trap) were used to estimate *Anopheles *densities. Every month, entomological sampling was carried out in two houses per village for two consecutive nights. The density of *Anopheles *per night per trap was calculated each month for each village by dividing the total number of mosquitoes caught by the total trap-night involved.

### Salivary peptide gSG6-P1

The gSG6-P1 peptide was designed using bioinformatics to maximize its *Anopheles *specificity and its antigenicity, as previously described [20]. The gSG6-P1 peptide was synthesized, purified (> 80%) by Genosys (Sigma-Genosys, Cambridge, UK) and biotin-conjugated (N-terminal). All peptides were shipped in lyophilized form and then resuspended in 0.22 μm ultra-filtered water and frozen in aliquots at -80°C until use.

### Evaluation of human IgG antibody levels (ELISA)

ELISAs were carried out on the sera to measure IgG Ab level reacting to the gSG6-P1 antigen. Maxisorp plates (Nunc, Roskilde, Denmark) were coated with gSG6-P1 (20 μg/mL) in carbonate/bicarbonate buffer. Individual sera (1:20) were incubated in PBS-Tween 1%. Anti-gSG6-P1 IgG detection was performed using an HRP goat anti-human IgG Ab (1:25,000, Nordic Immunology, Tilburg, Netherlands). Colorimetric development was carried out using ABTS (2,2'-azino-bis (3-ethylbenzthiazoline 6-sulfonic acid) diammonium; Sigma, St Louis, MO) in 50 mM citrate buffer (pH 4) containing 0.003% H_2_O_2_. Optical Density (OD) was measured at 405 nm. Each test sample was assessed in duplicate wells and, in parallel, in a blank well containing no antigen (ODn) to control for non-specific reactions in the plasma and the reagents. Individual results were expressed as ΔOD value calculated according to the formula ΔOD = ODx-ODn, where ODx represents the mean of individual OD in both antigen wells. The evolution of specific IgG anti gSG6-P1 was also investigated for each individual during the *Anopheles *exposure season. Results are expressed as ΔOD_season _value calculated according to the formula ΔOD_season _= ΔOD_December _- ΔOD_September_.

### Statistical analysis

All data were analysed with GraphPad Prism software^® ^(San Diego, CA, USA). After checking that the results did not have a normal distribution, the Wilcoxon matched pair test was used to compare paired sera from September and December and the non-parametric Kruskal-Wallis test was used for comparisons between more than two groups. All differences were considered significant at P < 0.05.

## Results

### Entomological and parasitological data

**Figure 1:**
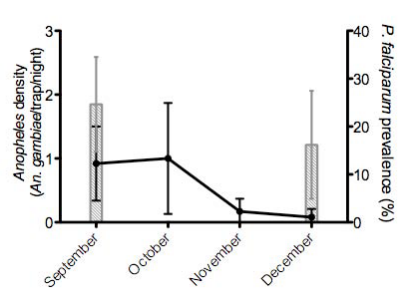
***An. gambiae *density and *P. falciparum *prevalence**. Continuous line indicates the level of exposure to *An. gambiae*, calculated by the arithmetic mean (± SD) of the number of *An. gambiae *collected per trap per night from September to December in the six villages. In September and in December, the columns indicate the *P. falciparum *prevalence in all children (n_September _= 403 and n_December _= 388) from studied villages.

In Niakhar area, more than 93% of the anopheline species belong to the *An. gambiae *complex as previously reported [15,21]. Entomological data based on collections from CDC traps indicated a similar pattern of *An. gambiae *density in the six villages during the studied period. The mean *Anopheles *density was very low in September (peak of exposure) and October to be absent in November and December (Figure 1). Indeed, 0 to 2.5 *Anopheles*/trap/night were only collected during the season and depending on village.

In contrast, a moderate prevalence of *P. falciparum *in all children (placebo group; 0-5 years old) living in these 6 villages was observed not only in September but also in December. In September, 24.8% of children presented a positive thick blood smear (from 11.1% to 41.5% depending on village). In December the prevalence decreased to 17.2% (from 9.3 to 33.9% depending on village). In addition, 17.6% of the children have developed a malaria attack during the exposure season (from 12.3% to 37.8% depending on village).

### Specific IgG responses to the gSG6-P1 peptide according to age group

**Figure 2:**
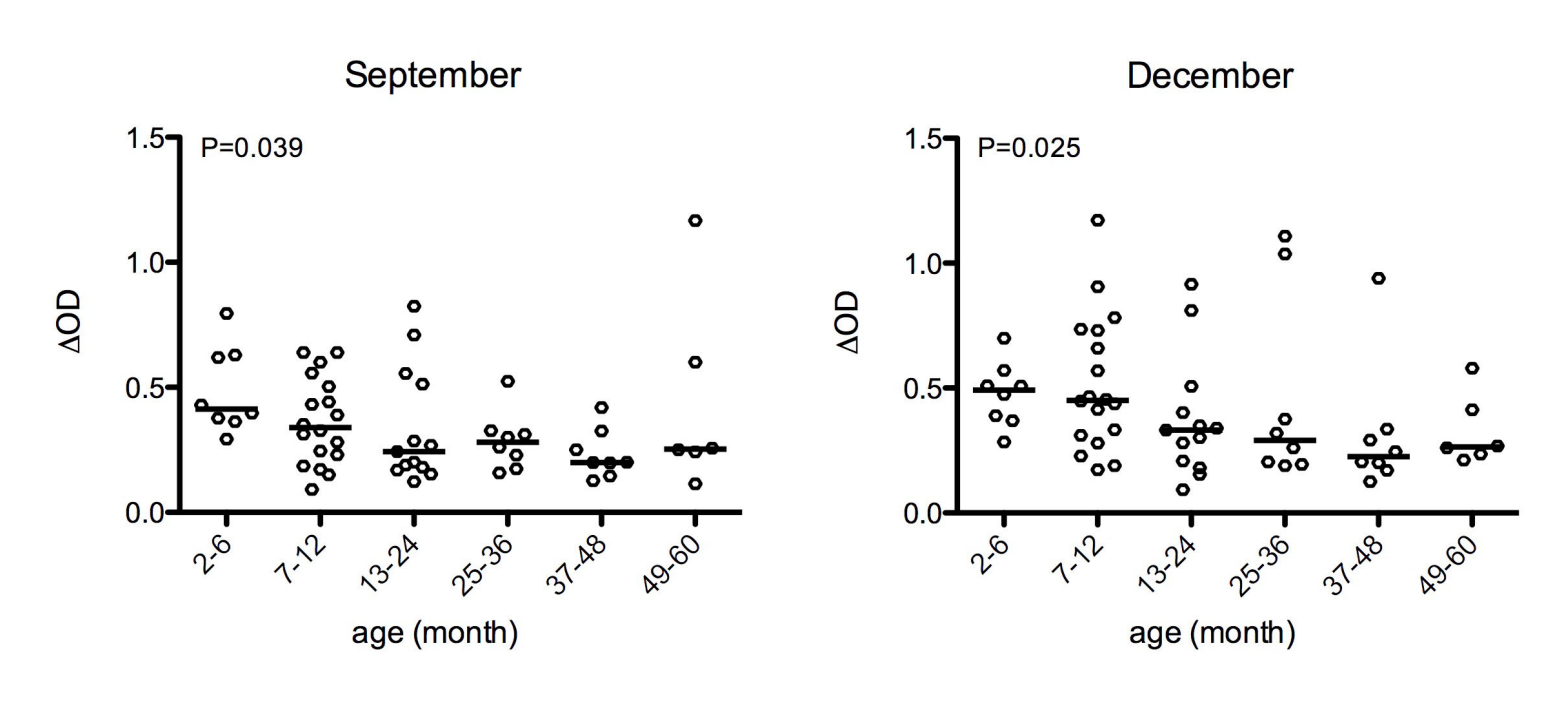
**IgG response to gSG6-P1 according to age groups**. Individual ΔOD in September and in December for children aged two to sixty months are presented according to age groups. Bars indicate the median value. Statistical significant differences between age groups are indicated (non-parametric Kruskal-Wallis test).

In a first analysis, IgG Ab levels specific to the gSG6-P1 peptide were evaluated according to the age of the children (sub-sample, n = 61) aged from two to sixty months at the peak (September) and at the end (December) of the season of *Anopheles *exposure. The median of IgG Ab level against gSG6-P1 differed significantly between age groups (< 1 to 5 years-old) both in September (P = 0.039) and in December (P = 0.025). In September, IgG responses were higher in the youngest children group (≤ 1 year-old), declining progressively through five years of age. A similar pattern of IgG level according to age group was observed in December (Figure 2).

### Specific IgG responses to the gSG6-P1 peptide during the exposure season to *Anopheles *bites at the population level

**Figure 3:**
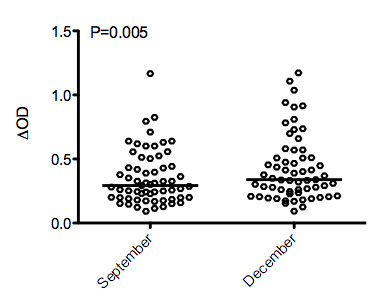
**Individual IgG antibody levels specific to *An. gambiae *gSG6-P1 in September and December**. Individual ΔOD results are presented and bars indicate the median value for each month. Statistical significant differences between months are indicated (Wilcoxon matched pair test).

The evolution of the specific IgG Ab response to gSG6-P1 was then analysed between September and December in the same children (Figure 3). In September, a considerable anti-gSG6-P1 IgG response was detected in most of individuals. In addition, the level of specific IgG Ab was significantly higher in December compared to September (P = 0.005) suggesting that the anti-gSG6-P1 IgG response increases during the exposure season.

### Specific IgG responses to the gSG6-P1 peptide during the exposure season to *Anopheles *bites at the individual level

The first analyse was at the population level. As a preliminary approach to establish a biomarker for individual exposure and behind the natural heterogeneity of individual exposure, the evolution of the anti-gSG6-P1 IgG response was individually evaluated for each pair of sera during the season of *Anopheles *exposure. For this purpose, the ΔOD_season _was defined and used to assess the individual trend (positive, negative or unchanged) between September and December.

**Figure 4:**
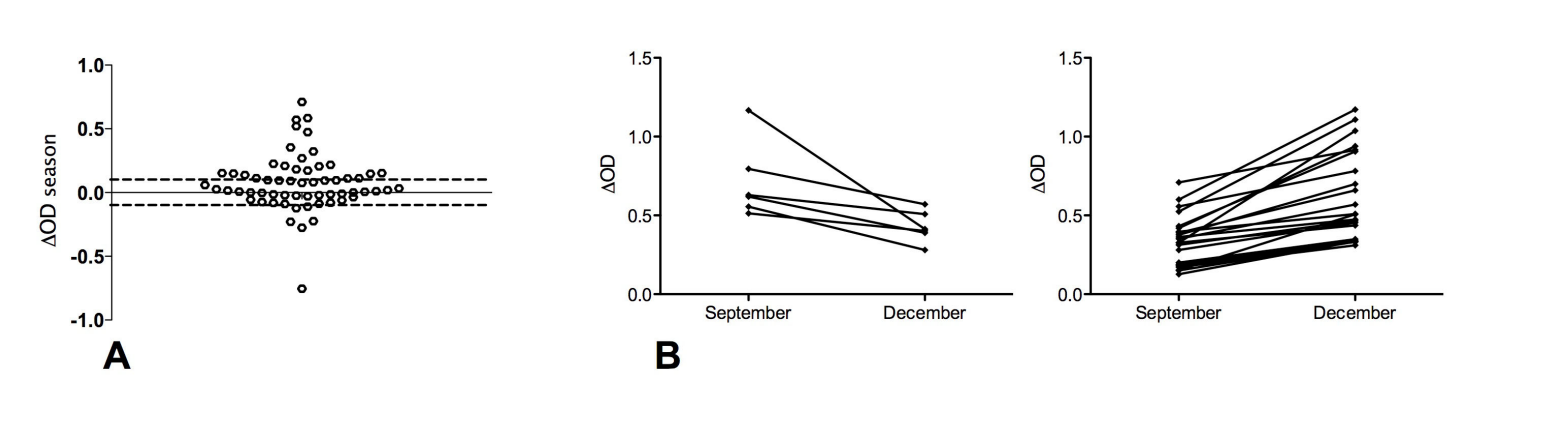
**Individual evolution of the IgG response to gSG6-P1 from September to December**. Individual ΔOD_season _of the IgG response specific to gSG6-P1 are presented (A). Individual evolutions of ΔOD from September to December are individually shown and positive (ΔOD_season _> 0.1) or negative (ΔOD_season _< -0.1) evolutions are separately presented (B).

Dissimilar evolutions were observed between individuals. Indeed, in applying an arbitrary threshold, 36% (22/61) of the children showed an increase of IgG specific to gSG6-P1 (ΔOD_season _> 0.1) and 10% (6/61) showed a decrease (ΔOD_season _< -0.1); 54% (33/61), showed no change (-0.1 < ΔOD_season _< 0.1) between September to December (Figure 4A). This result allows establishing a pertinent threshold to identify the children presenting an increase of specific Ab reponse. No significant difference was observed according to age groups in the IgG level evolutions (ΔOD_season_) between September to December (P = 0.129).

In addition, the individual evolution of the ΔOD can be assessed. Figure 4B shows individual ΔOD evolution from September to December from children presenting negative (down - ΔOD_season _< -0.1) or positive (up - ΔOD_season _> 0.1) evolutions.

## Discussion

This study focused on a particular and "paradoxal" malaria area, where *P. falciparum *transmission occurred in children during the exposure season, but where the classical entomological methods indicated that the exposure to *An. gambiae *was very low.

As it has been previously demonstrated that the specific IgG response to the salivary peptide gSG6-P1 was a biomarker of the exposure level to *An. gambiae *bites [20], the present study evaluated if the biomarker could be applied to detect a very low exposure to *An. gambiae *in children. The anti-gSG6-P1 IgG levels in the same children were followed from the peak (September) to the end (December) of the exposure season. This present study indicated that specific IgG response could be detected in children exposed to very rare bites in the same area of endemic malaria and presented an individual heterogeneity even if they live in the same area of exposure. In particular, specific IgG Ab were detected in most of exposed children and the IgG level was higher in December than in September whereas very few *An. gambiae *were collected over this period using classical entomological methods. Moreover, at the individual level, an increase in specific IgG Ab was clearly observed in 36% of children during the *Anopheles *exposure season (ΔOD_season _> 0.1). These results point to the potential of specific IgG responses to the gSG6-P1 peptide as an immuno-epidemiologic biomarker of exposure to *An. gambiae *bites, particularly in areas of very low exposure, where the sensitivity of current entomological methods is limited. In this particular context, it appeared also interesting that this biomarker could be used to evaluate the heterogeneity of individual exposure and therefore the individual risks of each child for malaria transmission.

The very low number of mosquitoes collected by the classical entomological methods in these specific areas can represent an inadequate or unreal measure of exposure. Indeed, it appeared clearly that children living in these villages were exposed to *Anopheles *vectors during the studied season. A considerable prevalence of *P. falciparum *infections was observed in these villages in September (11.1% to 41.5%) and also in December (mean = 17.2%) and 17,6% of children has developed one malaria attack from September to December. Altogether, these results indicated that an important malaria transmission occurred in these villages even if entomological methods did not indicate an exposure to *Anopheles *bites (in regards to few or no collected *An. gambiae*). This fact of malaria transmission indicate that children living in these villages have been well bitten by *Anopheles *vectors during the studied period, as the evaluation of anti-gSG6-P1 IgG response can suggest it.

Unfortunately, Ab responses had not been measured at the start of the rainy season (June, July), precluding analysis of the development of the responses from the start to the end of the *Anopheles *exposure season. In such areas, where less than 2.5 *Anopheles*/trap/night is collected between September and December (depending on village), an increase in IgG level specific to the gSG6-P1 is observed in 36% of children between the peak and the end of the exposure season (ΔOD_season _> 0.1). This could be due to a high sensitivity and specificity of the gSG6-P1 epitope(s) after a low immunological boost with just a few bites. Indeed, gSG6 protein was not a major immunogenic salivary protein according to immuno-blotting analyses in children exposed to moderate or high level of *Anopheles *[23] whereas specific IgG to this antigen has been reported in travellers who had only been exposed to *Anopheles *bites for a short period [19]. Furthermore, in the same area and period, a previous study had investigated the specific IgG response to whole *An. gambiae *saliva [15]. Children living in low exposure villages presented a significant decrease in anti-saliva IgG between September and December. In addition, previous results have demonstrated that the IgG response to another *An. gambiae *salivary protein (175 kDa) decreased during the exposure season in the same area [23]. Comparison of results from children living in the same area indicates that the Ab responses directed to whole saliva, to several salivary proteins or to the specific peptide differ according to the exposure intensity. It points to the immuno-sensitivity of the gSG6-P1 peptide and its suitability as a biomarker of low exposure to *An. gambiae *bites, in contrast to whole saliva or 175 kDa salivary proteins. In particular, the development of an anti-peptide IgG response could require repeated but rare *Anopheles *bites, in contrast to other proteins or whole saliva which only elicit a response after many bites, and which waning rapidly even with sustained exposure. Given the half-life of IgG antibodies (three weeks), the sustained anti-gSG6-P1 IgG response observed in some children could genuinely be due to repeated *Anopheles *bites throughout the studied period.

One major interest to evaluate the *An. gambiae *exposure by the approach of IgG response to gSG6-P1 is also the entire specificity to *Anopheles *bites. Indeed, the use of this peptide counteracts the possible cross-reactivity with shared epitopes on immunogenic salivary proteins from other arthropods, as previously described [20]. This specificity and sensitivity to the gSG6-P1 biomarker appeared to be especially efficient in a context of very low *Anopheles *exposure.

Interestingly, it has been reported that the highest specific IgG levels are seen in the youngest children, waning steadily through the first 60 months. This population of very young children (<12 months) is probably very weakly exposed to *An. gambiae *because of the protective behaviour of their mothers against mosquitoes (e.g. bednet protection during the night). Nevertheless, even the youngest children may be bitten by *Anopheles *although the high specific IgG response observed in this group could also result from the passive IgG transfer from mother to child during pregnancy or breastfeeding. It is known that maternal IgG persist in the infant blood for up to nine months after birth [24] and, at birth, foetal IgG typically somewhat exceeds maternal levels [25]. In this study, the acquired specific IgG response (induced by exposure to bites) and passive IgG transfer cannot be differentiate. This is currently being investigated. The important point when it comes to defining the validity of this exposure biomarker is that specific IgG responses to this peptide are observed in both very young and older children, the main population at risk of malaria.

In complement to the population approach, the individual IgG responses were investigated during the exposure season. In a low exposure context, few *Anopheles *are present and *An. gambiae *females tend to stay faithful to particular feeding sites (i.e. the female will return to the same house to feed thereby increasing clustering) [26]. These factors could increase the heterogeneity of the individual exposure risk in a given village. In this study, evolutions in specific IgG anti gSG6-P1 levels during the *Anopheles *exposure season seem to discriminate between those who are being bitten and those who are not.

## Conclusion

Taken together, these results indicated that measuring IgG level specific to gSG6-P1 during the exposure season could be used to evaluate a low level of exposure to *Anopheles *bites, notably at the individual level. This indicator, evaluated at just two time points, could be used to identify those who have been exposed to *An. gambiae *in very low exposure areas. This individual approach is complementary to entomological methods but contributes information about real human contact with anthropophilic *Anopheles*.

Such biomarker would be particularly relevant in places where malaria transmission is low, e.g. in foci of urban, high-altitude or seasonal malaria, and in travellers in endemic areas. In addition, this indicator of low exposure could be useful to monitor the risk of re-exposure to malaria vectors after a vector control campaign (insecticide, bednet) and could applied to other vector-borne diseases (e.g. *Aedes*-transmitted arboviruses).

## Competing interests

The authors declare that they have no competing interests.

## Authors' contributions

AP participated in the design of the study, carried out ELISAs, analysed and interpreted the data, and drafted the manuscript. SC contributed to the conception of the study and revised the manuscript. FB participated in the field study and performed entomological data. DB contributed to database management. CSow and MR performed the ELISAs. CSokhna and BC contributed to the field activities. FS participated in the conception of the study. FR conceived and coordinated the study and drafted the manuscript. All authors read and approved the final manuscript.
